# Complete Genome Sequence of *Algoriphagus* sp. Strain M8-2, Isolated from a Brackish Lake

**DOI:** 10.1128/genomeA.00347-16

**Published:** 2016-05-12

**Authors:** Yusuke Muraguchi, Koya Kushimoto, Yoshiyuki Ohtsubo, Tomohiro Suzuki, Hideo Dohra, Kazuhide Kimbara, Masaki Shintani

**Affiliations:** a Applied Chemistry and Biochemical Engineering Course, Department of Engineering, Graduate School of Integrated Science and Technology, Shizuoka University, Shizuoka, Japan; b Department of Environmental Life Sciences, Graduate School of Life Sciences, Tohoku University, Miyagi, Japan; c Research Institute of Green Science and Technology, Shizuoka University, Shizuoka, Japan; d Department of Bioscience, Graduate School of Science and Technology, Shizuoka University, Shizuoka, Japan

## Abstract

*Algoriphagus* sp. strain M8-2 was isolated from a brackish lake, Lake Sanaru, in Hamamatsu, Japan, as a filterable bacterium through a 0.22-µm-pore-size membrane filter. We report here the complete nucleotide sequence of the M8-2 genome (a 3,882,610-bp chromosome).

## GENOME ANNOUNCEMENT

*Algoriphagus* sp. strain M8-2 was isolated from a blackish lake, Lake Sanaru, in Hamamatsu, Shizuoka, Japan (137°40ʹ59ʺE, 34°42ʹ26ʺN). This strain was found and isolated on 28 April 2014 as one of the filterable bacteria from the lake. The strain M8-2 can form colonies on an agar plate even after filtration of the lake water through a 0.22-µm-pore-size membrane filter. It can grow on Difco marine broth 2216 with 1.5% agar at 30°C. The colony morphology on the medium was a circular form and convex elevation with a pink-orange pigment.

The genome of M8-2 was determined using MiSeq technology with a paired-end library (200,500 bp) prepared using the TruSeq DNA PCR-free library preparation kit (Illumina). A total of 2.0 million reads (479 Mb) preprocessed by ShortReadManager, which corresponds to a depth of 126-fold genome coverage, were used for assembly by the Newbler version 2.8 software (1). Finishing was conducted using the GenoFinisher, AceFileViewer, and GenomeMatcher softwares (2, 3), and gaps were closed by *in silico* analyses and PCR and Sanger sequencing of the PCR products. The finished sequence was confirmed by FinishChecker, which is an accessory tool of GenoFinisher.

The genome of M8-2 consists of a circular chromosome (3,882,610 bp; 41.4% G+C content). Sequence annotation was performed using the NCBI Prokaryotic Genome Annotation Pipeline (http://www.ncbi.nlm.nih.gov/genomes/static/Pipeline.html), and the resulting annotation was manually inspected with respect to the start codon positions using the Microbial Genome Annotation Pipeline (http://www.migap.org/), Prokka (4), and another annotation support tool of GenomeMatcher (2). The M8-2 chromosome has 3,377 coding sequences (CDSs), nine sets of rRNA genes, and 40 tRNA genes. M8-2 has the smallest genome (3.88 Mb) among the other sequenced genomes of the genus *Algoriphagus*, such as *A. boritolerans* (4.85 Mb) (DDBJ/EMBL/GenBank accession no. BBFN00000000), *A. machipongonensis* (4.89 Mb) (5), *A. mannitolivorans* (4.15 Mb) (6), *A. marincola* (HL-49, 4.18 Mb; DSM 16067, 4.43 Mb) (6), *A. terrigena* (5.78 Mb) (6), and *A. vanfongensis* (4.71 Mb) (6). A gene encoding putative proteorhodopsin was found on the M8-2 genome, showing homology with other proteorhodopsins of *Bacteroidetes*. Notably, sequenced strains with smaller-sized genomes, including those of *A. mannitolivorans*, *A. marincola*, and M8-2, possessed the putative proteorhodopsin genes.

### Nucleotide sequence accession number.

The nucleotide sequence of the M8-2 genome was deposited in the DDBJ/EMBL/GenBank databases under accession no. CP012836.

## References

[1] Wijaya E, Frith MC, Suzuki Y, Horton P 2009 Recount: expectation maximization based error correction tool for next generation sequencing data. Genome Inform 23:189–201. doi:10.1142/9781848165632_0018. 20180274

[2] Ohtsubo Y, Ikeda-Ohtsubo W, Nagata Y, Tsuda M 2008 GenomeMatcher: a graphical user interface for DNA sequence comparison. BMC Bioinformatics 9:376. doi:10.1186/1471-2105-9-376. 18793444 PMC2553346

[3] Ohtsubo Y, Maruyama F, Mitsui H, Nagata Y, Tsuda M 2012 Complete genome sequence of *Acidovorax* sp. strain KKS102, a polychlorinated-biphenyl degrader. J Bacteriol 194:6970–6971. doi:10.1128/JB.01848-12. 23209225 PMC3510582

[4] Seemann T 2014 Prokka: rapid prokaryotic genome annotation. Bioinformatics 30:2068–2069. doi:10.1093/bioinformatics/btu153. 24642063

[5] Alegado RA, Ferriera S, Nusbaum C, Young SK, Zeng Q, Imamovic A, Fairclough SR, King N 2011 Complete genome sequence of *Algoriphagus* sp. PR1, bacterial prey of a colony-forming choanoflagellate. J Bacteriol 193:1485–1486. doi:10.1128/JB.01421-10. 21183675 PMC3067637

[6] Tatusova T, Ciufo S, Fedorov B, O’Neill K, Tolstoy I 2014 RefSeq microbial genomes database: new representation and annotation strategy. Nucleic Acids Res 42:D553–D559. doi:10.1093/nar/gkt1274. 24316578 PMC3965038

